# *In silico* exploration of electric field distribution in tDCS: integrating white matter anisotropy and subject-specific structural connectivity

**DOI:** 10.3389/fnins.2026.1749851

**Published:** 2026-04-13

**Authors:** Giulia Caiani, Eleonora Arrigoni, Alberto Pisoni, Serena Fiocchi

**Affiliations:** 1 Dipartimento di Elettronica, Informazione e Bioingegneria, Politecnico di Milano, Milan, Italy; 2 Institute of Electronics, Computer and Telecommunication Engineering, National Research Council, Milan, Italy; 3 Department of Psychology, University of Milano-Bicocca, Milan, Italy

**Keywords:** computational modelling, DTI, structural connectome, tDCS, white matter anisotropy

## Abstract

Transcranial direct current stimulation (tDCS) is a non-invasive neuromodulation technique with promising application in the treatment of neurological and psychiatric disorders. However, its effectiveness is often limited by the high inter-subject variability of the induced effects, mainly attributable to individual anatomical differences, which are not considered in the design of the stimulation protocols. Among these, structural connectivity plays a crucial role but remains often overlooked in tDCS research. Objective—This study aims to evaluate how variations in structural connectivity influence the distribution of the electric field (EF) during tDCS session. In particular, we analyse how the inclusion of white matter anisotropy affects the EF distribution and spread compared to classical isotropic models, and how the strength of connection across cortical parcels affects the EF spread. Approach—The study proposes an advancement in the computational modelling of tDCS through the integration of white matter anisotropy into finite element method (FEM) simulations. By combining advanced computational approaches, we explore the relationship between EF strength and cortical connectivity. Main results—Neglecting white matter anisotropy in electromagnetic simulations lead to a relative error in EF magnitude greater than 10% and to an orientation error of the EF vector of almost 20 degrees. The DTI-informed simulations lead to a more focalized EF distribution, moreover it was found a positive and significant (*p* < 0.05) correlation between EF focality and the strength of connectivity between cortical areas below P2 and P1 electrodes. Significance—These findings highlight the importance of including white matter anisotropy into tDCS simulation to prevent distortions in EF distribution and suggest the need to integrate structural connectivity information into the definition of subject-specific dose in tDCS protocols.

## 1 Introduction

Transcranial Direct Cirrent Stimulation (tDCS) is a non-invasive brain stimulation technique that delivers a weak constant current through scalp-mounted electrodes (Nitsche and Paulus, 2000), generating electric fields (EF) that modulate neuronal excitability and induce synaptic plasticity (Liu et al., 2018). This mechanism has made tDCS a promising approach for enhancing sensorimotor functions and cognition in the treatment of a range of neuropsychological and psychiatric conditions, including depression, stroke rehabilitation and chronic pain (Kuo et al., 2014; Lefaucheur et al., 2017).

Despite this potential, clinical translation has been hindered by substantial inter-subject variability in aftereffects. A major source of this variability lies in the differences in EF distributions across individuals, which are strongly influenced by anatomical and physiological factors, such as skull thickness, cerebrospinal fluid distribution, scalp-to-cortex distance, gyral and sulcal morphology, age, and sex contribute (Evans et al., 2020; Laakso et al., 2019; Polanía et al., 2018; Guerra et al., 2020). Consequently, there is growing interest in developing personalized tDCS protocols that account for subject-specific neuroanatomy and physiology in order to increase reproducibility and efficacy (Evans et al., 2020).

Computational modelling approaches, which incorporate MRI-derived head models, have significantly improved the estimation of subject-specific EF distributions. However, most studies still rely on simplified assumptions such as isotropic conductivity for all tissue compartments, thereby neglecting the anisotropic nature of white matter (WM) (Saturnino et al., 2019).

WM is highly anisotropic due to its organized axonal architecture, which facilitates greater electrical conductivity along longitudinal fibres orientations than across them (Tuch et al., 2001). Diffusion tensor imaging (DTI) allows this anisotropy to be incorporated into computational models. Indeed, several studies have demonstrated that anisotropy can significantly alter local EF distributions, particularly within deep brain regions and along major fibre bundles (Suh et al., 2012; Abascal et al., 2008; Wagner et al., 2014; Windhoff et al., 2013). The integration of anisotropy in EF modelling has the potential to yield more biologically accurate prediction of current flow. Nonetheless, the magnitude and functional significance of these differences compared to isotropic models remain debated. Some studies suggest that anisotropy primarily modifies the topology of EF distribution rather than its global intensity, which may limit its relevance for standard stimulation paradigms (Huang et al., 2017; Opitz et al., 2015). In contrast, others highlight that neglecting WM anisotropy may overlook subject-specific features of structural connectivity that contribute to inter-individual differences in responsiveness (Bangera et al., 2010; Evans et al., 2023; Khan et al., 2022; Suh et al., 2010). From this perspective, anisotropic modeling becomes particularly important in precision neuromodulation, where capturing network-level effects and individual connectome properties is essential (Caulfield et al., 2020). Beyond its impact on neuromodulation, the integration of white matter anisotropy is equally critical for the inverse problem, where it significantly reduces errors in EEG dipole source localization with respect to isotropic models (Hallez et al., 2005).

Indeed, structural connectivity has been increasingly recognized as a critical determinant of variability in tDCS effects across individuals (Ferreri et al., 2017; Lin et al., 2017; Zhao et al., 2021). The organization and integrity of WM tracts influence how stimulation-induced fields propagate across cortical and subcortical regions, thereby shaping their functional impact. This highlights the need for computational models that integrate subject-specific connectome features with EF simulations. However, translating tractography reconstructions into reliable and biologically meaningful quantitative measures remains a considerable challenge. The streamline trajectories produced by diffusion tractography are mathematical abstractions rather than direct representations of axonal bundles (Maier-Hein et al., 2017). Among the various tractography-derived metrics, streamline count remains the most widely used to estimate connectivity strength between regions of interest (Hagmann et al., 2008). Yet this measure is highly sensitive to acquisition parameters, seeding strategies, and algorithmic biases, and does not reliably reflect underlying axonal density or cross-sectional area (Jones, 2010; Jones et al., 2013). To address these limitations, more advanced tractography frameworks have been developed to increase the biological plausibility of structural connectivity estimates. Spherical-deconvolution informed filtering of tractograms (SIFT, SIFT2) reweights streamlines to better match the fibre orientation distribution (FOD) derived from diffusion MRI, thereby improving the correspondence between streamline density and underlying axonal architecture (Smith et al., 2013, 2015). Other approaches, such as Convex Optimization Modeling for Microstructure Informed Tractography (COMMIT) (Daducci et al., 2015) and Linear Fascicle Evaluation (LiFE) (Pestilli et al., 2014), aim to prune or reweight streamlines by fitting them directly to the diffusion signal, reducing false positives and enhancing the interpretability of tractograms. Additionally, methods based on spin distribution functions, such as quantitative anisotropy (QA), have been proposed to mitigate the confounding effects of crossing fibres and increase robustness (Fang-Cheng Yeh et al., 2010). While these methods improve tractography-based connectome reconstruction, substantial limitations and lack of consensus on standardized methodologies that can lead to significant variability in connectivity estimates (Schilling et al., 2019) persist.

Addressing the overmentioned methodological challenges is crucial, as tractography-derived connectomes are increasingly integrated into clinical neuroscience, where biologically valid measures of connectivity are essential for understanding inter-individual variability and guiding personalized interventions. However, much of the existing literature exploring the inclusion of white matter anisotropy in computational modeling has been limited by small sample sizes, often relying on single-subject case studies or very small cohorts. This limits the generalizability of findings regarding the impact of anisotropy on EF distribution. Furthermore, while DTI remains the gold standard for implementing white matter anisotropy in current simulations, it suffers from a well-known inability to resolve crossing fibre populations, which can lead to anatomically inaccurate connectivity estimates in complex white matter regions (Fernandez-Miranda, 2013; Tallus et al., 2023).

Building on this premise, the present study aims to investigate how differences in structural connectivity influence the distribution of stimulation-induced electric fields, and to evaluate whether EF characteristics can be systematically related to tract-specific connectivity strength. This was performed in a cohort of thirty subjects, significantly increasing the statistical power and reliability of our results compared to previous studies. To achieve the overmentioned objectives, we combine finite element modeling with advanced diffusion MRI–based tractography frameworks that incorporate white matter anisotropy, providing a biologically informed approach to subject-specific modeling. By bridging EF simulations with quantitative measures of connectivity, our goal is to improve the accuracy of computational predictions and advance the development of precise and personalized neuromodulation strategies.

## 2 Materials and methods

### 2.1 Participants and data acquisition

#### 2.1.1 Participants

Thirty healthy, right-handed volunteers (seventeen males, mean age 23.4 years, SD 3.3, age range 19–34) participated in the study. All participants gave written informed consent prior to their involvement. The study was conducted in the RM3T laboratory of the University of Milano-Bicocca in accordance with the declaration of Helsinki and the approval of the local Ethics Committee (prot. N. 2024–812).

#### 2.1.2 Data acquisition

The anatomical T1-weighted MRI and diffusion-weighted (DW) images were acquired on a 3 T Philips Ingenia CX MRI scanner. T1w structural images were obtained using a 3D magnetization-prepared gradient-echo (MP-GRE) sequence (TR = 8.20 ms, TE = 3.79 ms, TI = 900 ms), with a 256 × 256 image matrix, 1 × 1 × 1 mm^3^ voxel size, and 117 echo train length. The DW images were acquired using a single-shot spin-echo (SE) echo-planar imaging (EPI) sequence (TR = 1815.34 ms, TE = 95.55 ms, slice thickness = 2.5 mm, acquisition matrix = 94 × 94, reconstruction matrix = 96 × 96). A multi-shell multi-tissue diffusion scheme was used, including 30 directions at *b* = 2000 s/mm^2^, 30 directions at *b* = 1,000 s/mm^2^, and 6 directions at *b* = 500 s/mm^2^, along with one *b* = 0 s/mm^2^ image. An additional b0 image with reversed phase-encoding (PE) direction was acquired. The primary PE was along the anterior–posterior direction, while the reversed PE acquisition was performed in the posterior–anterior direction. Multi-band acceleration was set to 2.

A total of 67 diffusion directions were acquired, based on the findings of Tournier and colleagues’ (Tournier et al., 2007), who demonstrated that at least 45 directions are required to achieve the highest angular resolution. Moreover, the maximum b values used in this study was set to 2000 s/mm^2^, as b-values exceeding 3,000 s/mm^2^ have been shown to lead to a reduction in signal-to-noise ratio (SNR) (Dietrich et al., 2008).

### 2.2 DWI preprocessing and whole brain tractography

Diffusion weighted image data were processed using an open-source software, MRtrix3 (https://www.mrtrix.org/) and FSL (https://fsl.fmrib.ox.ac.uk/fsl/docs/#/, FMRIB’s Diffusion Toolbox).

For each DWI, noise reduction (Veraart et al., 2016; Veraart et al., 2016) and Gibb’s ringing artefacts removal (Kellner et al., 2016) were performed. Then b0 images acquired in both PE and reversed PE direction were used for EPI-distortion (Holland et al., 2010), B0-field inhomogeneity (Andersson et al., 2003; Smith et al., 2004; Schenck, 1996), eddy-current and movement distortion correction (Andersson and Sotiropoulos, 2016; Le Bihan et al., 2006).

To find the direction of the white matter fibers from the preprocessed DWI, firs the response function of the CSF, white and grey matter, were estimate through the *dhollander* algorithm (Dhollander et al., 2021). Fiber orientation distributions (FODs) were then estimated in each voxel through multi-shell, multi-tissue constrained spherical deconvolution (Tournier et al., 2007; Tournier et al., 2004). The use of diffusion weighted images with multiple b-values helped to overcome the challenge of crossing fibers.

A probabilistic algorithm was employed to identify white matter tracts together with the anatomically constrained tractography (ACT), to increase the biological plausibility of the reconstructed streamlines (Smith et al., 2012).

To further address the non-quantitative nature of diffusion MRI tractography, the SIFT2 method was applied. This approach refines streamline reconstructions by assigning an appropriate cross-sectional area multiplier to each streamline, enabling biologically accurate measures of fiber connectivity while preserving the complete tractogram (Smith et al., 2013; 2015).

Together with the previously reported steps, several strategies were considered to ensure comparability of diffusion data across subjects. First, a global inter-subject intensity normalization of pre-processed DWIs was performed using the *median white matter b = 0 intensity*. Second, constrained spherical deconvolution (CSD) for each subject was performed using the same average response function for each tissue, computed across all thirty subjects. Finally, fiber orientations were normalized through a multi-tissue informed intensity normalization.

### 2.3 Structural connectivity and ROIs definition

For each subject a whole-brain connectome was generated based on the HCP MMP 1.0 atlas, Human Connectome Project Multi-Modal Parcellation 1.0 (Glasser et al., 2016), which comprehends 180 parcels for each hemisphere. The parcellation was performed using Freesurfer software (https://surfer.nmr.mgh.harvard.edu/).

The structural connectivity (SC) matrix of each subject was computed through the MRtrix function *tck2connectome*. Each value of the matrix represents the number of streamlines connecting the two nodes, weighted by the inverse of the volumes of the corresponding parcels, to account for differences in node size across the dataset (Byrne et al., 2024).

The SC matrix allows to gain quantitative information on the strength of connection between cortical regions.

To reduce the computational costs, the investigations were focused on four regions of interest (ROIs), selected according to the cortical parcels mostly connected to the anodal region. First, the atlas parcels corresponding to the area under the anode (P2) were identified, roughly corresponding to the right posterior parietal cortex (rPPC). This work is based on the same protocol followed by the Romero Lauro and colleagues’ study (Romero Lauro et al., 2014), in which the anode was placed over the rPPC and the cathode on the contralateral supraorbital area. This montage provides anodal stimulation to the PPC, cortical target region to modulate sensorimotor and cognitive processing in healthy participants (Ikkai and Curtis, 2011; Fogassi and Luppino, 2005), but also to enhance the performance in tasks requiring visuospatial attention, often compromised in various neurological and psychiatric conditions (Wright and Krekelberg, 2014; Roy et al., 2015).

**Figure 1:**
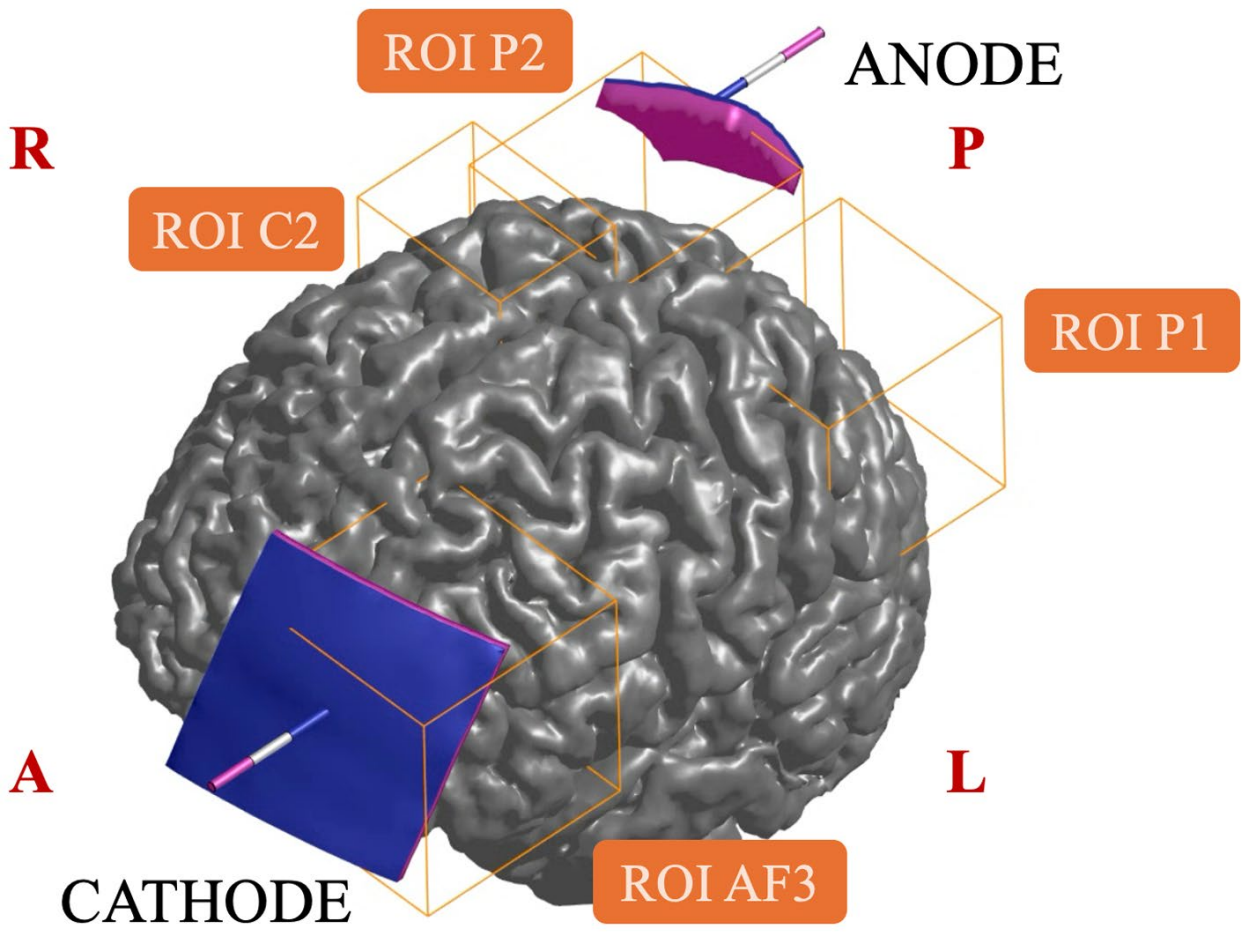
tDCS montage (anode over P2 and cathode over AF3) and region of interest (ROIs) from which EF distributions were extracted. A, anterior; P, posterior; R, right; L, left.

Next, to determine the most connected parcels to this region, all cortical parcels from the HCP MMP 1.0 atlas were ranked in descending order based on their total number of structural connections (streamline count) to the P2 seed region. The top 15 highest-ranked parcels were then evaluated within both the ipsilateral and contralateral hemispheres, and two cortical regions were identified: the one below the C2 (i.e., label anatomica) and P1 (i.e., label anatomica) electrodes according to the 10–10 EEG system (Figure 1). These two ROIs plus the two ROIs corresponding to the region under the anode and the cathode (AF3) were then used to construct a custom-made atlas, from which reduced 4×4 structural connectivity matrices were derived using MRtrix.

**Figure 2:**
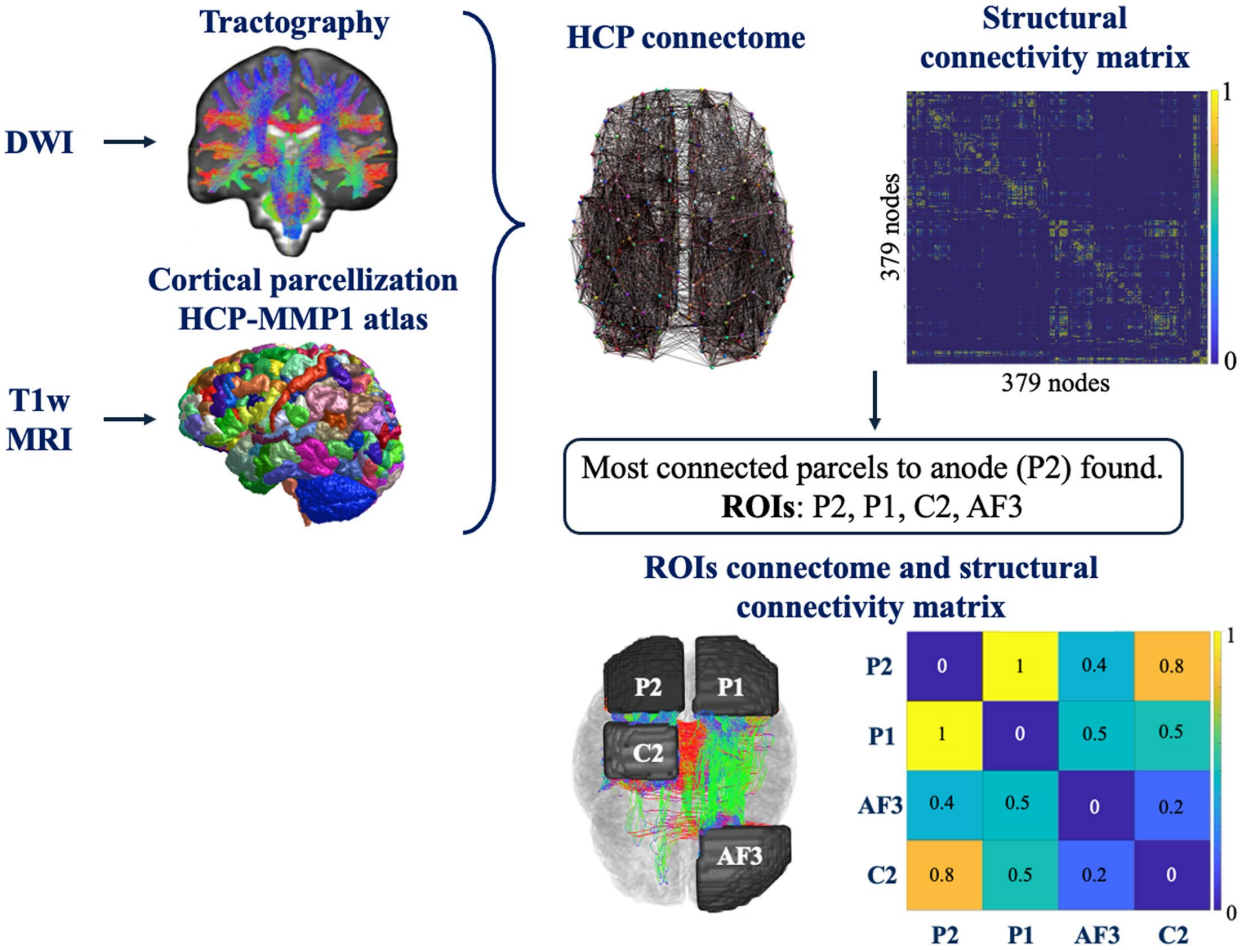
Workflow of the extraction of the structural connectome from DWI and T1w MRI for each subject using MRtrix, FSL, and FreeSurfer.

The workflow for the two kind of structural connectivity matrices construction is showed in Figure 2.

### 2.4 Anatomical modelling

Simulations were performed on subject-specific head models, reconstructed in a voxel-based format by the segmentation of high resolution (i.e., 1 mm) T1-weighted structural MRI. The segmentation was performed using the Sim4Life (ZMT Zurich MedTech AG, 2024) *eHead40* function, which allows to distinguish up to forty different tissues at the head level - including skin, CSF (both external and ventricles), bone cortical, bone cancellous, brain grey and white matter and internal air. To balance tissue reconstruction accuracy with computation time, the output voxel spacing was set to 0.3 mm.

### 2.5 EM characterization and simulation settings

tDCS simulations were performed using the electromagnetic commercial software Sim4Life (ZMT Zurich MedTech AG, 2024). In the near DC frequency range relevant to tDCS, the quasi-static Laplace Equation 1 is considered as a valid approximation (Filmer et al., 2020; Parazzini et al., 2012; Ridding and Ziemann, 2010) to determine the electric potential distribution ( φ ) inside the human models due to stimulation:


∇(σ∇φ)=0 (1)

where *σ* (S/m) is the electrical conductivity of the tissues. For electrically anisotropic materials such as white matter the conductivity can be represented by a symmetric 3 × 3 tensor as shown in Equation 2:


$$\sigma =(\begin{array}{ccc} \sigma_{\mathrm{xx}} & \sigma_{\mathrm{xy}} & \sigma_{\mathrm{xz}}\\ \sigma_{\mathrm{yx}} & \sigma_{\mathrm{yy}} & \sigma_{\mathrm{yz}}\\ \sigma_{\mathrm{zx}} & \sigma_{\mathrm{zy}} & \sigma_{\mathrm{zz}} \end{array})$$ (2)

The EF distribution in each point of the conductive medium was obtained by means of the Equation 3:


EF=−∇φ (3)

For each participant two simulations were performed: one considering all tissues isotropic and another one including white matter anisotropy (in the following named “NoDTI-Sim,” “DTI-Sim”).

The conductivities of the head tissues were assigned according to the data collected in the IT’IS low-frequency tissue properties database (Hasgall et al., 2022). The isotropic electrical properties of the tissues mostly involved in tDCS (Saturnino et al., 2019) are: *σ_skin_* = 0.148 S/m, *σ_fat_* = 0.078 S/m, *σ_CSF_* = 1.879 S/m, *σ_grey matter_* = 0.419 S/m, *σ_white matter_* = 0.348 S/m, *σ_bone cancellous_* = 0.08 S/m, *σ_bone cortical_* = 0.0063 S/m.

The white matter tissue anisotropy was assigned based on the hypothesis that the orientation of the diffusion tensor major eigenvector, derived from DWI, is generally assumed to be parallel to the local white matter bundles (Basser et al., 1994; Alexander et al., 2007). The dedicated Sim4Life pipeline was utilized for the integration of DWI data in the simulations, specifically through the *s4l-dti* python package (https://github.com/dyollb/s4l-dti). First, the diffusion tensor data were reconstructed from the DWI preprocessed in MRtrix, then the reconstructed diffusion tensors were converted into a conductivity tensor field applying the effective medium approach as described in Tuch and colleagues’ study (Tuch et al., 2001), assigned voxel-wise to the white matter tissue. This ensures that the simulated current flow accounts for both the inhomogeneity (voxel-specific variations) and the anisotropy (directional dependence) of the white matter.

In all simulations electrodes were placed according to the 10–10 EEG system: the anode placed over the posterior parietal cortex and the cathode over the contralateral supraorbital area, in P2 and AF3, respectively. The electrodes were modelled as rectangular pads (3 × 3 cm^2^, 5 × 5 cm^2^ for anode and cathode, respectively) of 1 mm thick copper (*σ* = 5.9 × 10^7^ S/m) placed above a conductive sponge [*σ* = 1.4 S/m (Datta et al., 2011)] with the same dimensions and thickness of 5 mm (Caiani et al., 2025). The modelling was done though SimNIBS v4.1.0 (https://simnibs.github.io/) (Nielsen et al., 2023) and then meshes of both copper and sponge were smoothed in Meshmixer (Autodesk, Inc. v11.2.37). This integrated procedure ensures a highly reproducible framework for the electrodes configuration, optimizing their modelling and reducing the uncertainty of their placement.

The two electrodes were set to a fixed potential (+/− 1 V) and the results were later scaled to simulate a current of 0.75 mA, consistent to the fixed-dose tDCS approach used in Romero and colleagues’ study (Romero Lauro et al., 2014). This specific intensity was selected as it represents a balanced compromise between ensuring a sufficient dosage to elicit a physiological response and maintaining the low current levels approved by the local Ethical Committee.

The computational domain was truncated at the neck level to reduce computational cost. The resulting domain was of 65 × 40 × 40 cm^3^. A non-uniform hexahedral mesh was used for discretization, with cell resolutions ranging from 0.5 mm for brain tissues and electrodes to 2 mm in other regions. This high resolution was specifically chosen to mitigate the staircasing error, known to be a numerical artifact in voxel-based models that occurs when curved tissue boundaries are approximated with rectilinear grids. Increasing the resolution of the model makes the simulations’ results less sensitive to the hexahedral mesh (Laakso and Hirata, 2012). This approach ensured a fine representation of small structures, such as the skin, while maintaining computational efficiency. The final mesh consisted of approximately 110 million cells.

### 2.6 Analysed quantities

#### 2.6.1 Structural connectivity

The structural connectivity matrix was extracted from each subject’s DWI. Subsequently, the following metrics were evaluated:

- Adjusted interindividual variability in brain connectomes. It was evaluated using the following Equation 4 (Mueller et al., 2013):

$$\mathrm{Var}(i)=1-E[\text{corr}(SC_{i}(s_{p}),SC_{i}(s_{q}))]$$ (4)

where SC_i_ is the structural connectivity between region i and all the other regions, s_q_ and sp. refers to subjects (p,q = 1, 2,…, N p ≠ q) (Suh et al., 2012)

- Ranking of the parcels mostly connected to the cortical area under the anode among all the parcels of the atlas.
- Ranking of the parcels mostly connected to the cortical area under the anode among the parcels in the left hemisphere.

The cortical area under the anode (PPC) was considered as the one composed by the following parcels of the HCP-MMP1 atlas all in the right hemisphere (R_): MIP, PCV, 7Pm, PGs, 31pd, 31a, IPS1, POS2, V7, 7PC, 7AL, VIP, 7Am, 7PL, LIPv, LIPd, AIP, V6A, IP1, DVT.

#### 2.6.2 EF distribution

Once the parcels most connected to the anode were identified, the EF distribution on both the white and grey matter of the whole brain and on four specific regions of interest (ROIs, Figure 1) was extracted. The EF was calculated as a vector average of the EF in a small contiguous tissue volume of 2 mm^3^ × 2 mm^3^ × 2 mm^3^, as a compromise that balances the need of a robust biological basis with computational feasibility (International Commission on Non-Ionizing Radiation Protection, 2010; Fiocchi et al., 2016). This spatial averaging allows also to limit numerical errors (Soldati and Laakso, 2020).

To investigate the intersubject variability in structural connectome and the influence of white matter anisotropy in the amplitude, spread and orientation of the EF, the following metrics were extracted or computed:

- MaxEF: Peak amplitude of the EF distribution in white matter across all ROIs.
- MaxDiff: Peak difference in EF distribution in white matter between NoDTI-Sim and DTI-Sim.
- RE (Residual Error): Equation 5 quantifies the relative difference between EF distribution in white matter in NoDTI-Sim and DTI-Sim (Suh et al., 2012):

$$\mathrm{RE}=\sqrt{\frac{\sum_{i=1}^{N}{\left(EF_{\text{NoDTI}}(i)-EF_{\mathrm{DTI}}(i)\right)}^{2}}{\sum_{i=1}^{N}{\left(EF_{\mathrm{DTI}}\right)}^{2}}}\;$$ (5)

- V50, V70, V80: percentage volume of the brain (white and grey matter together) where the EF amplitude was greater than the 50, 70% of the MaxEF for each ROI. They assess the effect of DTI-based modelling on tDCS focusing capability.
- MaxAlpha: Peak of the angle difference between EF orientations in white matter in NoDTI-Sim and DTI-Sim, found following Equation 6 (Parazzini et al., 2017):

$$\text{Alph}a_{\left(\mathrm{DTI}-\text{NoDTI}\right)}(i)=\cos^{-1}(\frac{{\vec{\mathrm{EF}}}_{\mathrm{DTI}}(i)\cdot {\vec{\mathrm{EF}}}_{\text{NoDTI}}(i)}{{\vec{\mathrm{EF}}}_{\mathrm{DTI}}(i){\vec{\mathrm{EF}}}_{\text{NoDTI}}(i)})$$ (6)

MaxEF, MaxDiff, MaxAlpha are computed using the 99th percentile of the EF distributions to filter possible spurious points due to numerical errors (Fiocchi et al., 2016).

#### 2.6.3 Correlations

To assess how the strength of connections between the anode area and the other three regions of interest affects the electric field quantities correlations between reduced structural connectivity matrixes and EF quantities were quantified. The correlations were obtained through the Pearson correlation coefficient *r* and its correspondent *p*-value. The closer to +/−1 the stronger the correlation is (Soliani Lamberto, 2008). If the p-value is under the significance level of 0.05 the correlation found can be considered significant. The p-value is obtained through testing the hypothesis that there is no relationship between the variables (null hypothesis) (Press et al., 1992; Kendall, 1979; Fisher, 1958). The Pearson coefficient was deemed appropriate as the measures involved in the study, both the structural connectivity matrices and the EF quantities, are intrinsically linked to anatomical characteristics, which are typically normally distributed in the general population (Żytkowski et al., 2021). Moreover, with a sample size of *N* = 30, Pearson’s *r* provides sufficient sensitivity to evaluate the linear relationship between analysed quantities. The whole analysis was performed in MATLAB (Version 2024a, https://www.mathworks.com/products/).

## 3 Results

### 3.1 Structural connectivity HCP-MMP1 atlas

**Figure 3:**
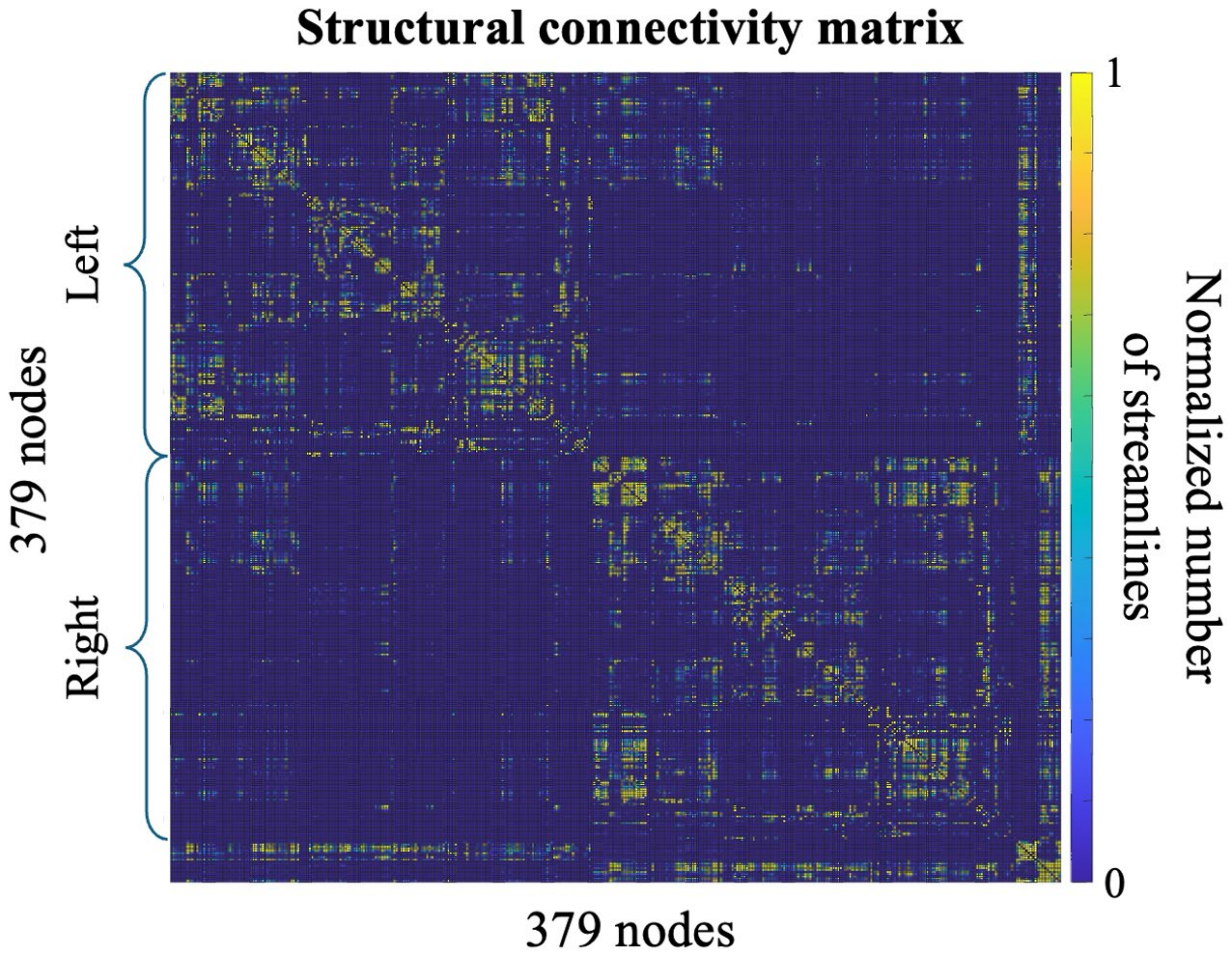
Structural connectivity matrix showing the connections among the 379 parcels defined by the HCP-MMP1 parcellation. The matrix values, expressed as the number of streamlines, are normalized between 0 and 1.

SC matrixes found following the HCP-MMP1 atlas (Figure 3) are visually consistent with previous studies (Tsai, 2018; Rosen and Halgren, 2021). The median of the adjusted intersubject variability across all brain parcels is 37.9%, with an interquartile range of 0.6%.

**Figure 4:**
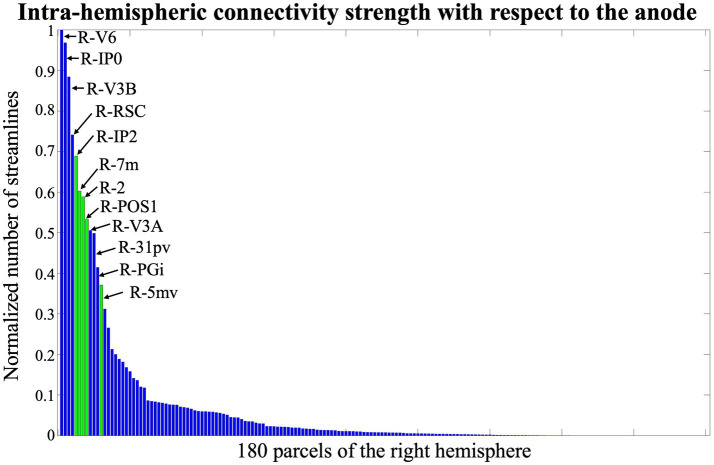
Normalized number of streamlines between the anode and right-hemisphere parcels, sorted by connectivity strength. Green bars correspond to parcels beneath the C2 position of the 10–10 EEG system.

**Figure 5:**
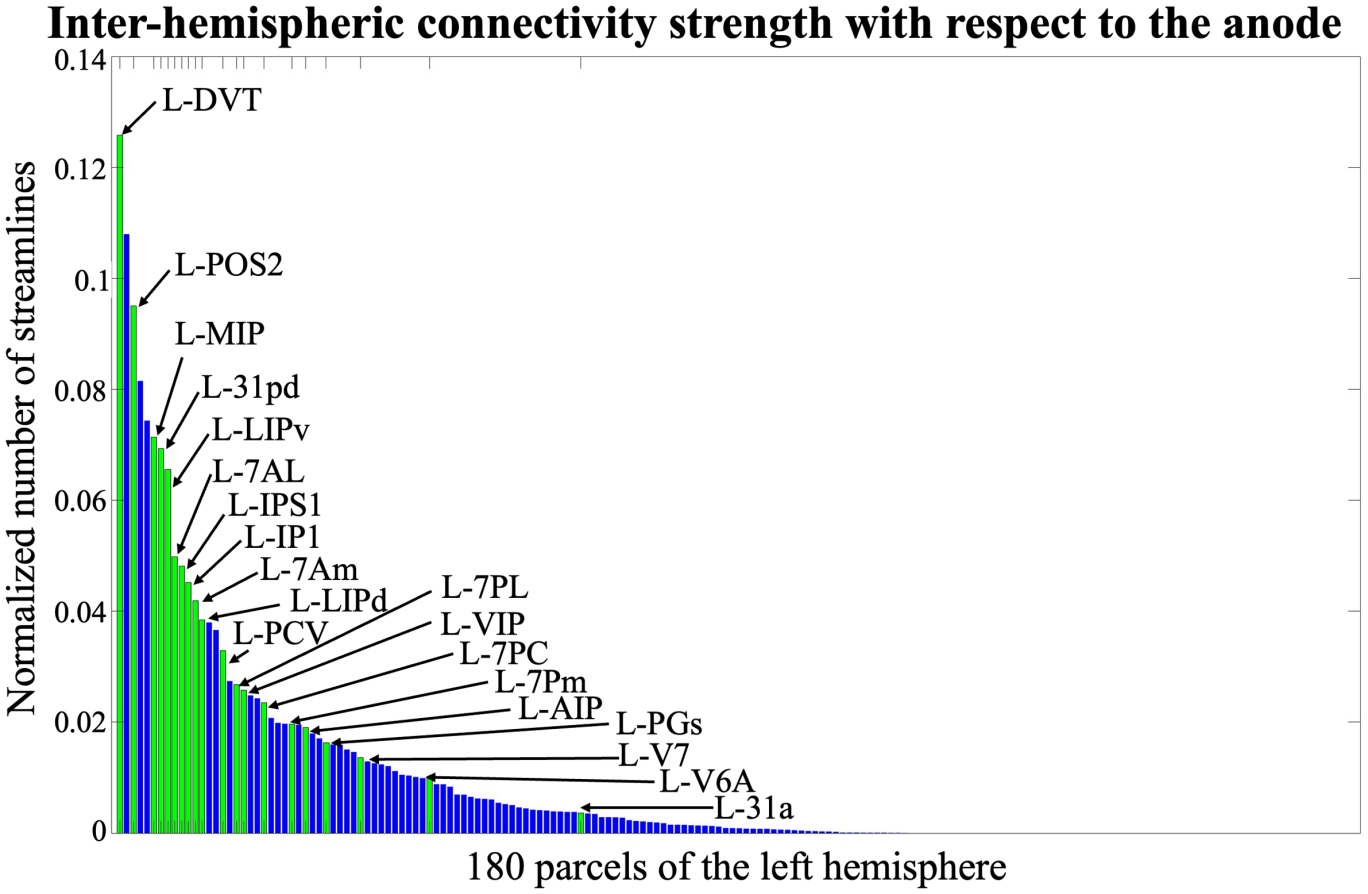
Normalized number of streamlines between the anode and left-hemisphere parcels, sorted by connectivity strength. Green bars correspond to parcels beneath the P1 position of the 10–10 EEG system.

The highest variability, i.e., 38.9%, is reached in the limbic associative areas and in the auditory association area, while the lowest variability, i.e., 5%, is found in parcels of the visual and primary motor cortex. The inter-subject variability of connections between the parcels under the anode and all the other regions, is 37.7%. Despite the observed variability, we identified two key regions - one homolateral and one contralateral to the anode - comprising the parcels with the strongest structural connections to the anode area. In the right hemisphere, the most strongly connected parcels are in the upper part of the precentral and postcentral cortex, beneath the C2 position in the 10–10 EEG reference system (Figure 4). In the left hemisphere, the most connected parcels correspond to the homologous regions of those under the anode, positioned near the P1 reference point (Figure 5). This rationale guided the volume of interest for EF analysis, centering them in P2, C2, and P1. Additionally, AF3 was included as it corresponds to the cathode placement, despite the absence of direct structural connections between its parcels and those under the anode.

### 3.2 Impact of DTI in EF distribution

**Figure 6:**
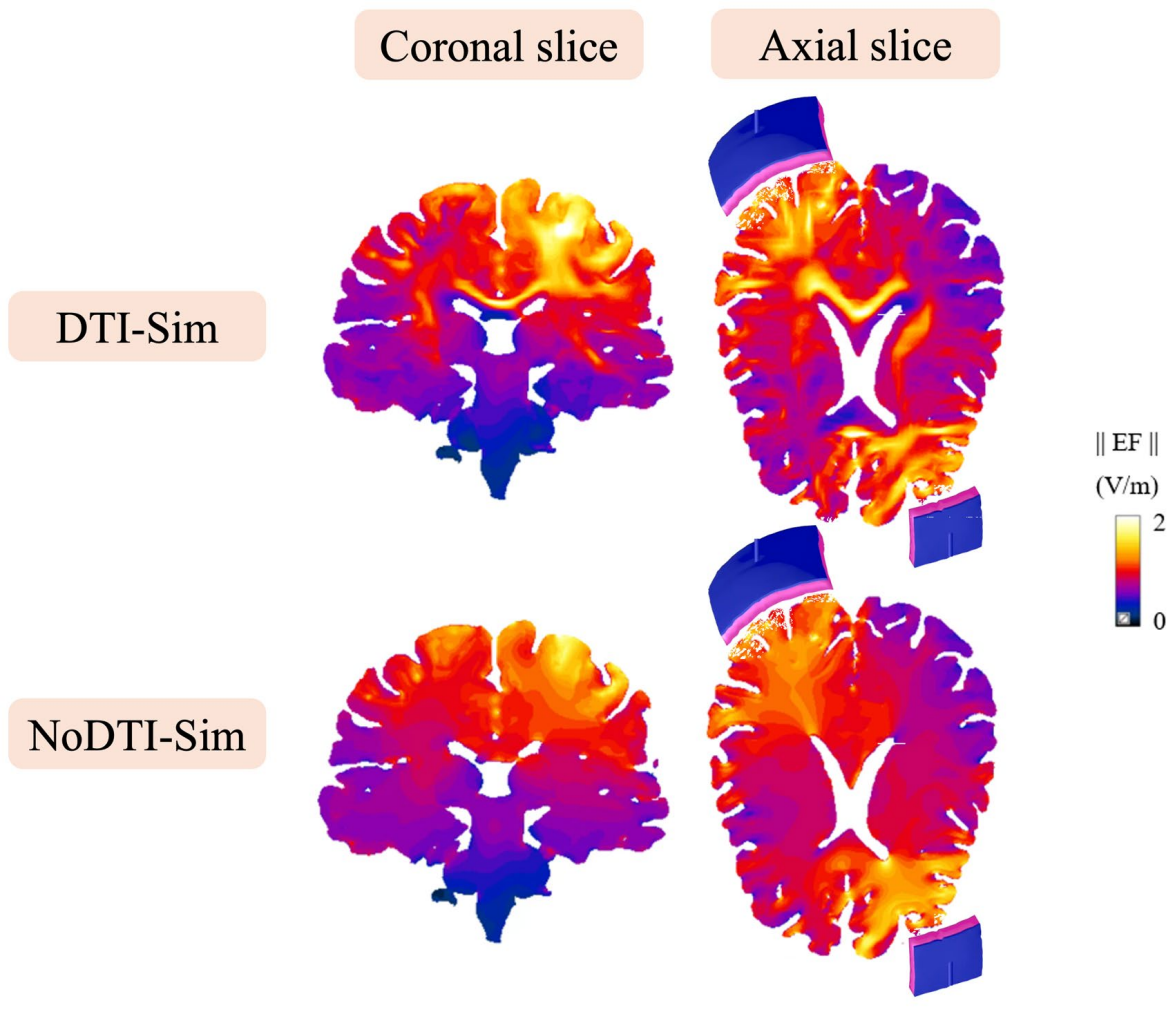
EF distribution on coronal and axial slices of white and grey matter for DTI and NoDTI simulations. The axial view shows the position of the stimulation electrodes (anode and cathode). Results are shown for a representative subject.

Figure 6 shows the differences in EF distribution when white matter anisotropy is either included or neglected in the simulations for a representative subject. A clear local EF enhancement (e.g., in corpus callosum) is observed in regions where the current is forced to cross fibre pathways in an orthogonal projection. This effect arises due to a reduction in conductivity along these pathways (Shahid et al., 2014). Similar spatial patterns of EF distribution were consistently observed across all 30 subjects.

Table 1 quantifies the differences in EF distribution with and without white matter anisotropy. Notably, the inclusion of DTI reveals higher EF values, with the largest EF difference in C2 and P2 (under the anode). Specifically, the EF difference between the two models reaches 46% relative to the peak EF in the isotropic simulation. These results highlight the significant impact of neglecting white matter anisotropy, leading to potential errors in estimating the EF distribution. To systematically assess this discrepancy, the RE for each ROI (AF3, C2, P1, P2) was reported. The values obtained indicate that the error remains consistently above 10% across all ROIs.

**Table 1:** EF distribution in NoDTI and DTI simulations in each ROI.

ROIs	MaxEF [mV/m]		MaxDiff [mV/m]	Relative error [%]	MaxAngle [deg]
	NoDTI	DTI			
P2	191.5 ± 83.1	199.5 ± 88.7	71.2 ± 35.6	12.3 ± 5.4	24.0 ± 10.5
P1	123.0 ± 52.6	125.8 ± 53.9	36.2 ± 17.8	9.7 ± 4.2	24.2 ± 10.5
AF3	167.4 ± 72.0	158.5 ± 68.4	40.4 ± 21.5	12.4 ± 5.5	23 ± 9.9
C2	169.2 ± 72.7	211.3 ± 93.9	78.1 ± 40.3	13.2 ± 5.8	25.1 ± 10.7

Value expressed as mean and standard deviation.

### 3.3 Impact of DTI in EF orientation and spread

Beyond magnitude differences, the directionality of the EF is also affected by neglecting anisotropy. Our analysis reveals that ignoring DTI-based anisotropy introduces an orientation error of approximately more than 20 degrees, specific values are reported in Table 1 (MaxAngle).

The spread of the EF in each ROI was further analysed using V50, V70, and V80 metrics. Across the entire brain volume, including both grey and white matter, we observed a greater EF focalization when DTI is included, values are reported in Table 2.

**Table 2:** EF spread in NoDTI and DTI simulations in each ROI.

ROIs	V50 [%]		V70 [%]		V80 [%]	
	NoDTI	DTI	NoDTI	DTI	NoDTI	DTI
P2	32.7 ± 6.9	31.1 ± 7.3	10.7 ± 4.1	10.3 ± 3.4	3.9 ± 1.4	4.1 ± 1.4
P1	34.5 ± 5.7	33.5 ± 5.7	15.7 ± 4.5	14.6 ± 3.6	5.6 ± 2.0	5.2 ± 1.4
AF3	41.9 ± 7.6	41.6 ± 7.5	22.5 ± 5.2	17.7 ± 4.4	7.0 ± 2.1	6.2 ± 1.5
C2	68.2 ± 5.3	59.9 ± 6.3	25.2 ± 6.8	17.5 ± 4.3	9.2 ± 2.7	6.6 ± 1.7
Brain	21.9 ± 1.6	18.9 ± 1.7	6.6 ± 0.7	5.3 ± 0.7	2.8 ± 0.3	2.3 ± 0.3

Value expressed as mean and standard deviation.

These results indicate that incorporating white matter anisotropy leads to a more localized EF distribution. When examining individual ROIs, this focalization effect is most evident in C2, which is the region most structurally connected to the anode. Despite reaching the highest MaxEF, C2 exhibits also the strongest EF focused effect due to anisotropy.

### 3.4 Linking EF quantities to structural connectivity ROI-based atlas

**Figure 7:**
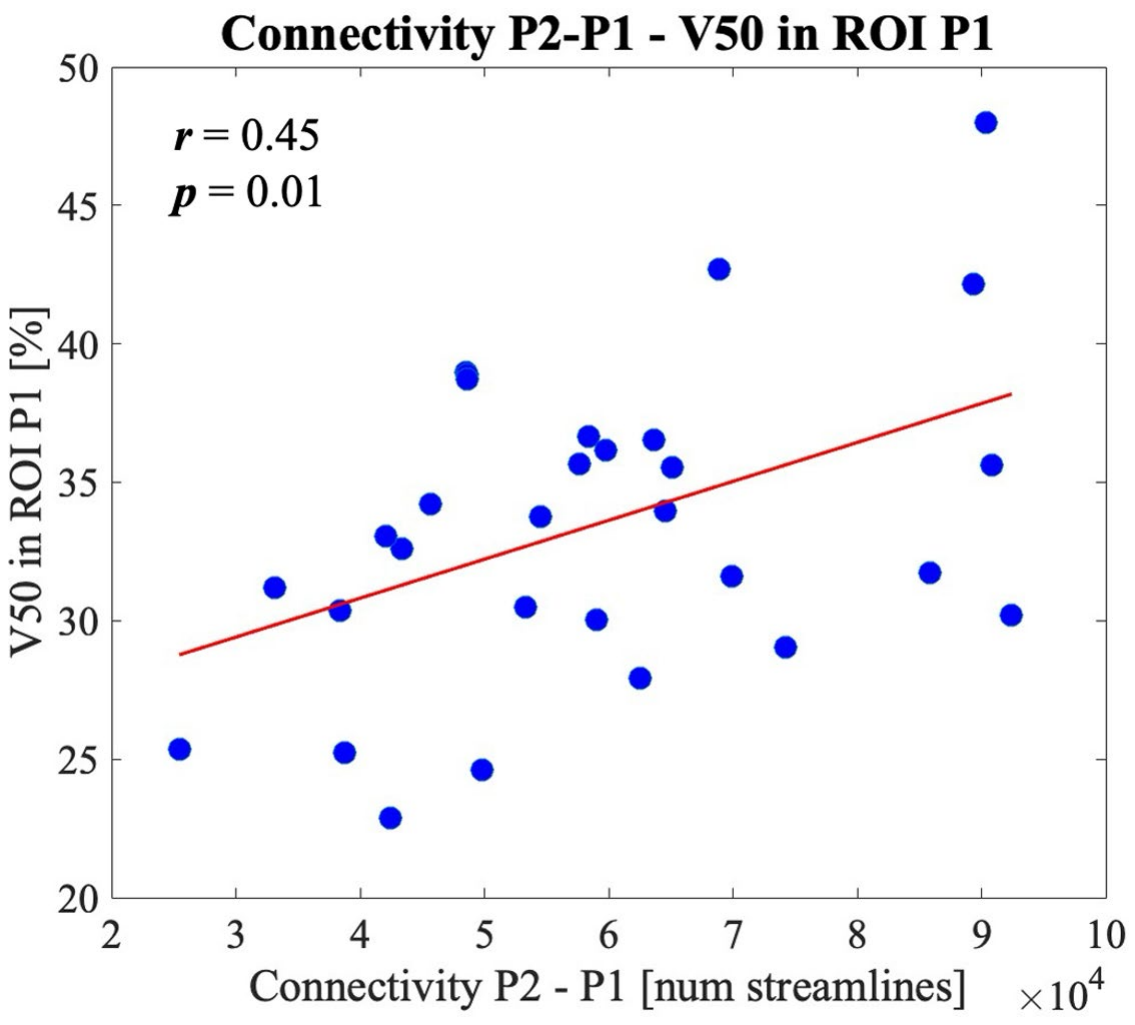
V50 linear behaviour with respect to the connectivity strength between cortical area under the anode and the contralateral area P1. With V50 being a measurement of spread of the electric field in the ROI P1.

Among all the EF quantities investigated our analysis revealed a positive correlation between V50 in the DTI simulations and the strength of connections between ROI P2-P1 (*r* = 0.45, *p* = 0.01), as shown in Figure 7. Weaker correlations, even if not statistically significant, exist also between V50 and connections P2-AF3 and P2-C2.

## 4 Discussion

Transcranial direct current stimulation (tDCS) is a promising non-invasive neuromodulation technique capable of modulating cortical excitability through the injection of a weak current (Nitsche et al., 2003). Despite this, its use is still hampered by the lack of individualized injected dose, not taking into account anatomical characteristics of subjects (Evans et al., 2020).

Previous computational studies addressed this issue by modelling the electric field distribution induced during tDCS sessions. However most of them assumed isotropic conductivity of brain tissues, which may lead to a biased estimation of current propagation.

In this study, we propose to address this research gap by integrating DTI information into our model to investigate how differences in structural connectivity affect the distribution of stimulation-induced electric fields, and whether EF quantities can be systematically related to tract-specific connectivity strength.

The structural connectivity (SC) matrixes (Figure 3) found in this work are consistent with previous findings of studies using probabilistic tractography (Tsai, 2018; Rosen and Halgren, 2021). The regions showing lowest and highest variability of connections align with the findings of (Huang et al., 2025) who reported that SC shows greater variability in limbic regions while lower in unimodal sensorimotor regions. In particular, the high variability (37.7%) of the connection of the brain beneath the anode and the other parcels emphasizes the need to account for these differences when interpreting results for the specific stimulation setup used in our study.

The inclusion of DWI-derived anisotropy in the model resulted in higher EF magnitudes (Table 1). The ROIs showing the greatest differences are P2 and C2, which are the two parcels mostly connected within the right hemisphere. The relative error (RE) analysis shows the sistematical discrepancy between isotropic and anisotropic models, exceeding 10% across all ROIs. These results highlight the significant impact of neglecting white matter anisotropy, leading to potential errors in estimating the EF distribution (Miranda et al., 2018; Samani et al., 2017).

DTI-informed model showed a slightly more focal electric field across the whole brain volume, including white and grey matter (Table 2). The region of interest mainly affected by this focalizing effect was C2, which is also the one more strongly connected to the anode within the same hemisphere. This focalization is directly attributable to anisotropy, which channels the current preferentially along white matter tracts (Shahid et al., 2013).

Moreover Table 1 show also than an orientation error of more than 20 degrees can be found when ignoring anisotropy. This is particularly critical for microscopic neural analysis, where even subtle changes in EF orientation can influence subthreshold neuronal responses (Foerster et al., 2019). To reduce the possible impact of the artifact related to the staircase effect at tissue interfaces, common issue in voxel-based meshes (Laakso and Hirata, 2012), a high-resolution discretization (0.5 mm for brain tissues) was employed in the computational model, together with an appropriate post-processing of the EF. In particular, the application of spatial averaging over a 2 × 2 × 2 mm^3^ volume, combined with the exclusion of values above the 99th percentile, significantly reduces spurious peaks and numerical artifacts arising at tissue interfaces (Soldati and Laakso, 2020).

The influence of structural connectivity across the selected regions of interest on EF quantities was evaluated using correlation analyses (Figure 7). We found that the stronger the connectivity between a ROI and the stimulation target (anode), the greater the percentage of brain tissue exposed to higher electric field magnitudes. This suggests that regions with stronger structural connections experience a more focused EF, particularly those located near the anode, such as the P1 area.

The cortical area beneath P1 (left PPC), implicated in somatosensory integration, visuospatial processing, multisensory association, and higher-order cognition (Whitlock, 2017), represents a desirable target for multiple tDCS applications (Bjekić et al., 2019; Heimrath et al., 2012; Zivanovic et al., 2021). Nonetheless, depending on the stimulation montage and desired outcome, EF propagation toward contralateral or functionally connected regions (e.g., from P2) could be detrimental or introduce unwanted effects. Thus, the directionality and spread of the EF must be carefully considered when selecting stimulation targets. The increased EF focalization observed in this region suggests that DTI-informed simulations could improve the precision and efficacy of such interventions. In this case as well, our findings highlight the relevance of incorporating individual structural connectivity when planning stimulation, to ensure consistent and targeted engagement of functionally relevant areas.

While this study focused on one specific electrodes montage, it is known that different configurations, in terms of number, placement and distance of the electrodes, significantly affect the distribution and orientation of the electric field within cortical regions (Richardson et al., 2015). We acknowledge that the impact of the inclusion of anisotropy in simulations may vary depending on the placement of the anode, which is the one conceiving the higher values of EF. For instance, in regions where WM anisotropy is less pronounced, the deviation of the EF might be smaller. However, our results show that even in a standard two-electrode montage, ignoring anisotropy leads to a significant misrepresentation of the EF distribution. The assessment of the impact of different configurations of electrodes is out of the scope of this study, but the computational framework developed is highly adaptable. Future research could leverage this pipeline to investigate the impact of structural connectivity on more complex arrangements, such as High-Definition (HD-tDCS) configurations.

The observed correlations between EF metrics and the strength of connections among the investigated ROIs are particularly relevant in light of our previous studies (Caiani et al., 2025), where we demonstrated how the inter-subject differences in anatomical characteristics significantly modulate the variability of tDCS after-effects. These after-effects, evaluated through TMS-evoked potentials (Romero Lauro et al., 2014), were found to be strongly dependent on the EF spread parameter V50, which plays a crucial role in defining the individualized injected current.

The present finding that V50 is affected by the inclusion of white matter anisotropy in the simulations and its positive correlation with the strength of connection between the anode area and the contralateral region, further supports the need to personalize stimulation parameters based not only on anatomical features but also on structural connectivity.

Moreover, the variability across subjects in terms of structural connectivity matrices likely contributes to the heterogeneity of tDCS after-effects observed across subjects and should be considered alongside with other anatomical determinants, such as cerebrospinal fluid volume (Caiani et al., 2025).

Altogether, these findings open the way for future investigations into how connectivity-informed tDCS protocols could be optimized for personalized neuromodulation strategies.

## 5 Conclusion

tDCS is a promising non-invasive neuromodulation technique; however, its efficacy is often limited by high variability in aftereffects, largely due to the absence of protocols that account for individual anatomical differences when determining stimulation parameters.

While in literature there are few studies accounting for how structural connectivity differences can affect cortical responces of patients undergoing neuromodulation techniques (Giannakakis et al., 2020; Lin et al., 2017), as far as we are concerned this is one of the first studies underscoring the importance of integrating personalized protocols that consider white matter connectivity during the tDCS optimization process. Metrics reflecting the strength of connections between the anode and other cortical areas should be incorporated into stimulation design to better account for the observed variability in electric field distribution. Incorporating DTI data results in increased intensity and focality of the electric field, particularly in regions structurally connected to the anode, highlighting the potential of using white matter connectivity information to achieve more precise and effective neuromodulation. The focus was mainly on the regions connected to the anode, since anodal stimulation, in contrast to inhibitory cathodal stimulation, is typically associated with an increase in cortical excitability (depolarization), and therefore represents the most promising tDCS treatment option (Brückner and Kammer, 2017; Elsner et al., 2020).

A key innovation of this work is that it not only systematically demonstrates the differences between simulations with and without DTI information, but also examines how structural connectivity, in terms of connection strength, influences the EF, with a particular focus on spread metrics. As shown in previous studies, these metrics are critical for predicting cortical responses to stimulation.

Overall, our results provide a foundation for future research on the personalization of tDCS protocols based on brain connectivity, aiming to develop more effective, targeted, and individualized neuromodulation strategies tailored to each subject’s brain architecture.

## Data Availability

The datasets presented in this study can be found in online repositories. The repositories can be found here: https://osf.io/n7fpg/overview?view_only=d45ba3c6989748e692fdf9f12011e99b.
